# Supplementary material for: BRCA1 deficiency specific base substitution mutagenesis is dependent on translesion synthesis and regulated by 53BP1
Source: Nat Commun. 2022 Jan 11;13:226. doi: 10.1038/s41467-021-27872-7 (PMC8752635; doi:10.1038/s41467-021-27872-7)
Supplement: Supplementary file 1 — Supplementary Information [file 41467_2021_27872_MOESM1_ESM.pdf]

SUPPLEMENTARY INFORMATION

Title

BRCA1 deficiency specific base substitution mutagenesis is dependent on translesion synthesis and regulated by 53BP1

Authors

Dan Chen[1], Judit Z. Gervai[1], Ádám Póti[1], Eszter Németh[1], Zoltán Szeltner[1], Bernadett Szikriszt[1], Zsolt Gyüre[1,2], Judit Zámborszky[1], Marta Ceccon[3], Fabrizio d'Adda di Fagagna[3, 4], Zoltan Szallasi[5, 6, 7], Andrea L. Richardson[8], Dávid Szüts[1]

[1] Institute of Enzymology, Research Centre for Natural Sciences, Budapest, H-1117 Hungary
[2] Doctoral School of Molecular Medicine, Semmelweis University, Budapest, H-1085 Hungary
[3] IFOM Foundation-FIRC Institute of Molecular Oncology Foundation, Via Adamello 16, 20139, Milan, Italy
[4] Istituto di Genetica Molecolare, Consiglio Nazionale delle Ricerche (IGM-CNR), Via Abbiategrasso 207, 27100, Pavia, Italy
[5] Computational Health Informatics Program (CHIP), Boston Children's Hospital and Harvard Medical School, Boston, MA 02215, USA
[6] Danish Cancer Society Research Center, Copenhagen, 2100 Denmark
[7] SE-NAP, Brain Metastasis Research Group, 2nd Department of Pathology, Semmelweis University, Budapest, H-1092 Hungary
[8] Johns Hopkins University School of Medicine, Baltimore, MD 21287, USA

Contents

Supplementary Figures 1-6

Supplementary Tables 1-3

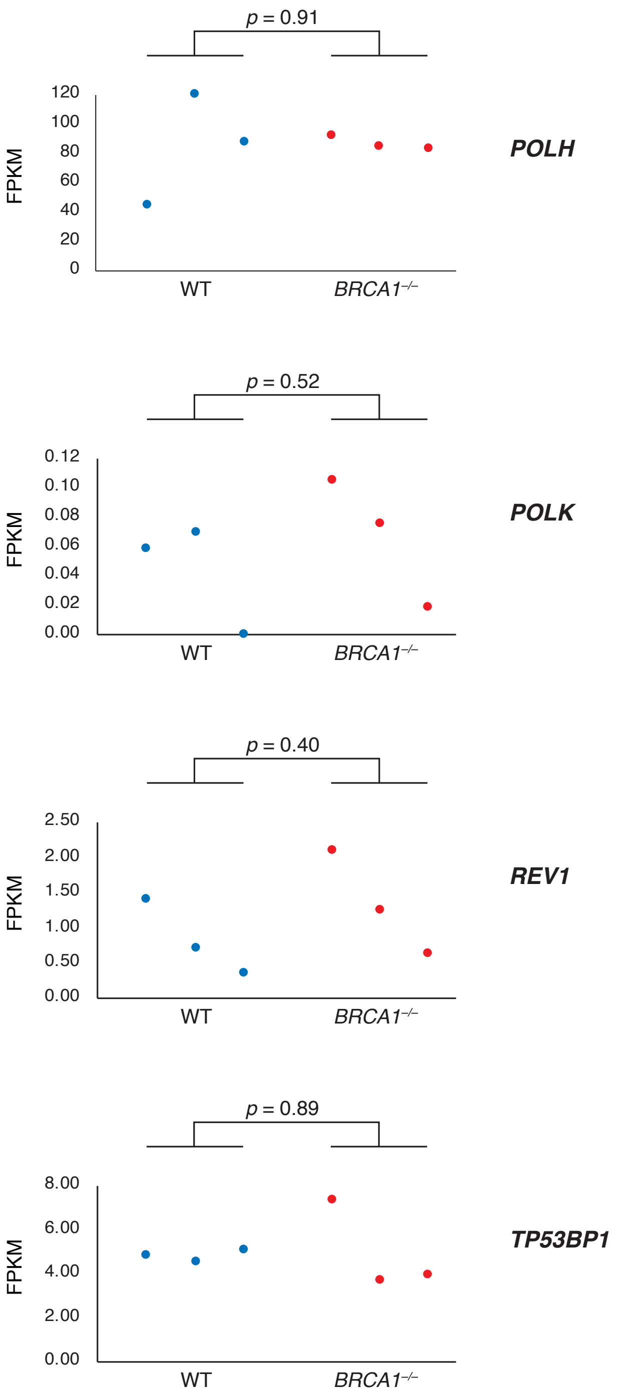

Supplementary Figure 1. Expression of TLS genes and *53BP1* in WT and *BRCA1*$^{-/-}$ cells.

Expression level of the indicated translesion polymerase genes and the *53BP1 (TP53BP1)* gene in three independent samples derived from a wild type (WT) or a *BRCA1*$^{-/-}$ culture, assayed by next generation sequencing of total RNA. Values are shown as fragments per kilobase transcript per million reads (FPKM). Significance values derived from unpaired two-sided *t*-tests are shown on each panel.

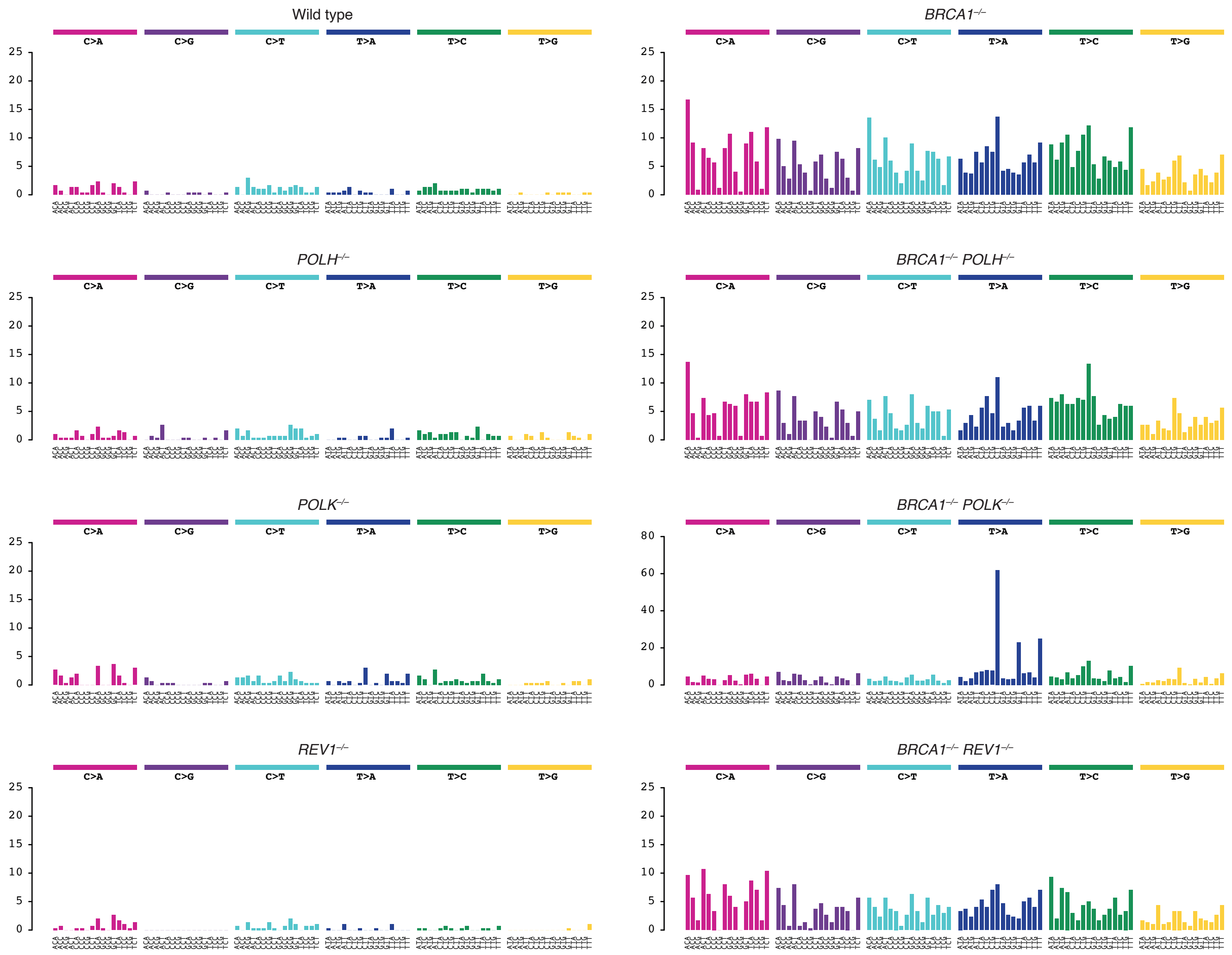

Supplementary Figure 2. Spontaneous triplet mutation spectra of cell lines deficient for BRCA1 or TLS polymerases.

Spontaneous triplet mutation spectra of DT40 cell lines of the indicated genotypes. Base triplets are shown below the spectra; the base substitution at the central position is colour coded and shown above. The *y* axis showns the average number of mutations per genome that arose over 50 days.

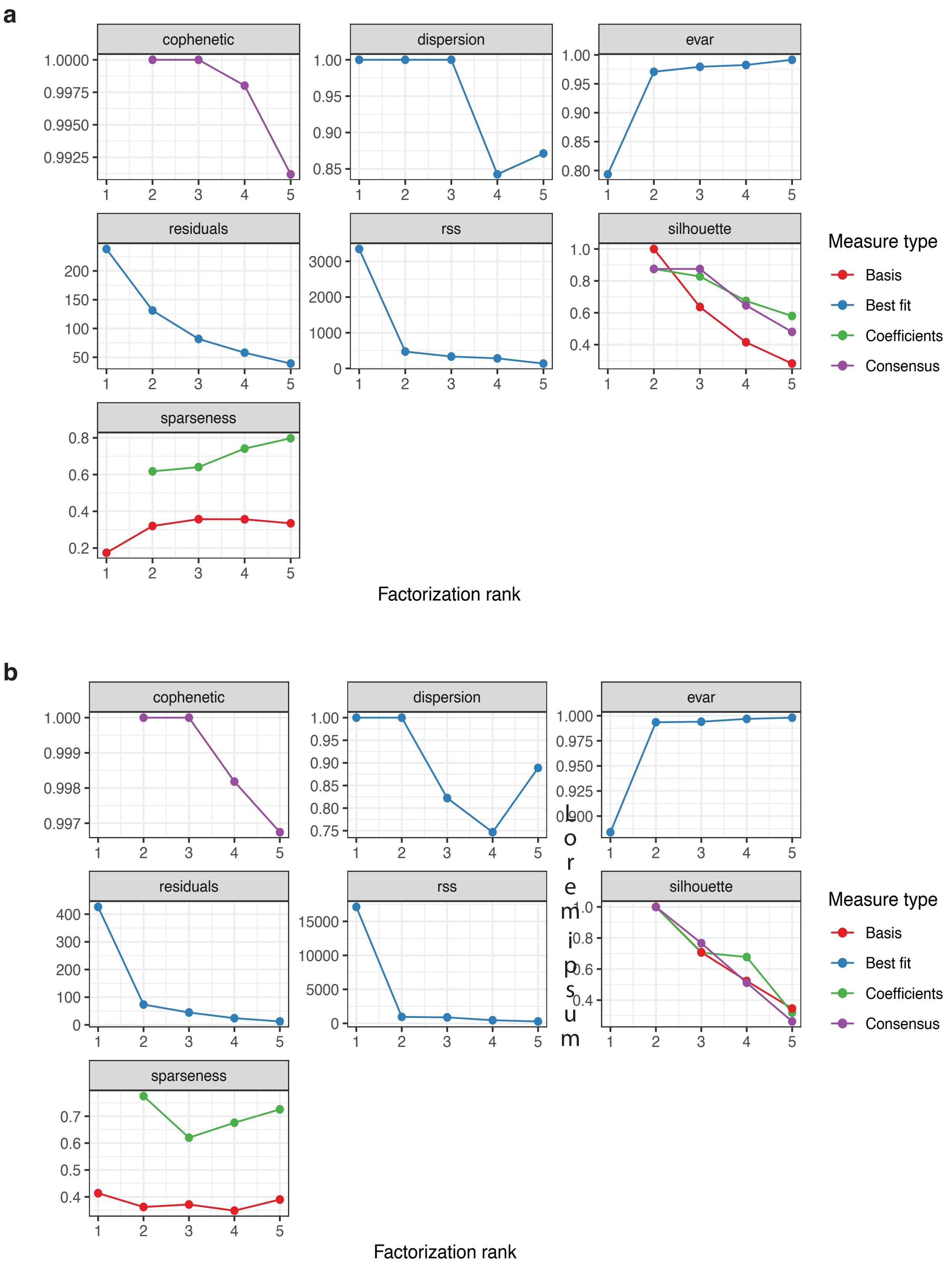

Supplementary Figure 3. NMF rank survey.

Non-negative matrix factorisation rank survey for deriving the SNS mutational signatures presented in (**a**) Figure 1 and (**b**) Figure 2, from the R package *MutationalPatterns*. A factorisation rank of 3 was chosen in each case based on the cophenetic correlation coefficient values.

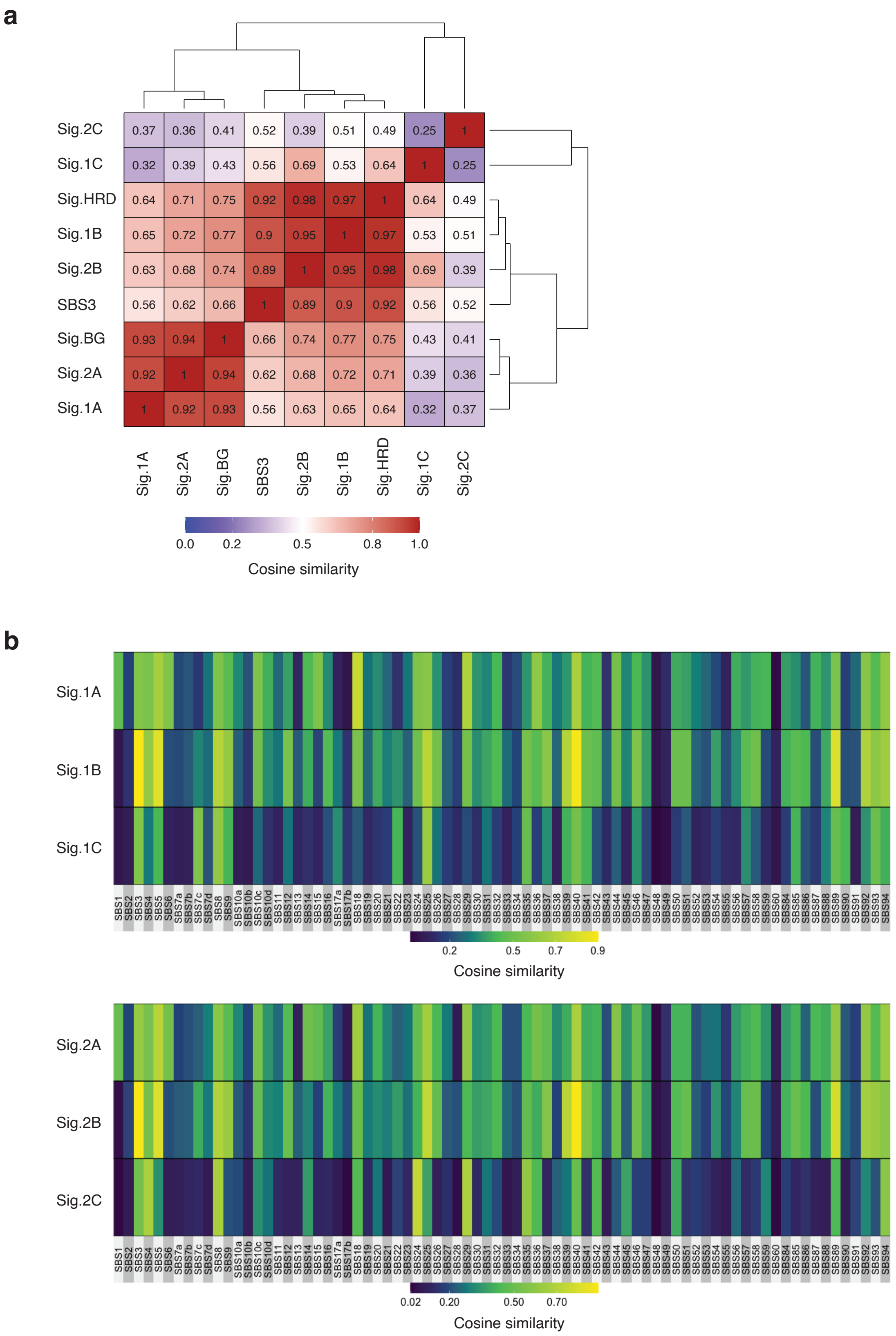

Supplementary Figure 4. Comparisons of base substitution signatures.
a Hierarchical clustering and pairwise cosine similarity of mutational signatures extracted in this work (Sig.1A, Sig.1B, Sig.1C, Sig.2A, Sig.2B, Sig.2C), the 'background' and 'homologous recombination deficiency' signatures identified in reference 8 (Sig.BG, Sig.HRD) and COSMIC v3.2 signature SBS3. **b** Heat map of cosine similarities of the newly extracted signatures, normalised to the frequencies of triplet occurrence in the human genome, to all non-artefactual SBS signatures in COSMIC v3.2.

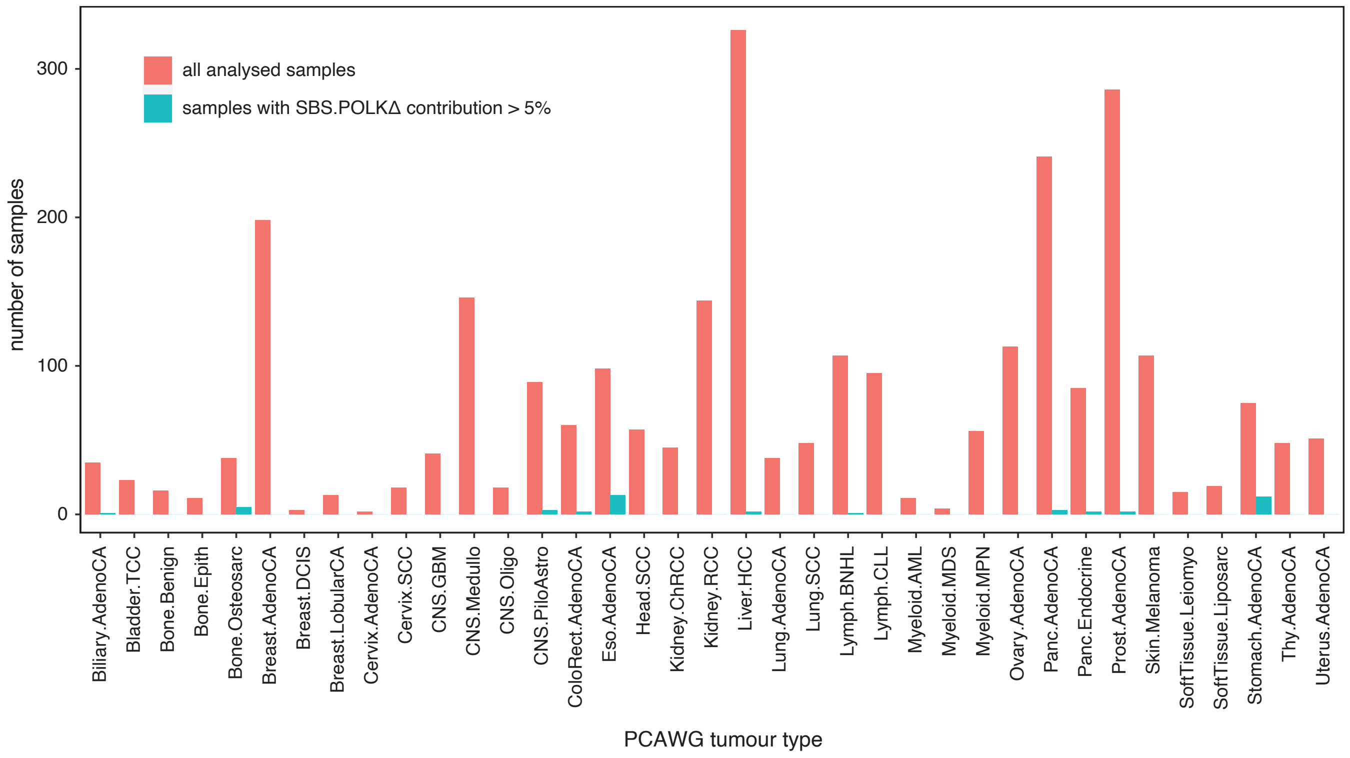

Supplementary Figure 5. The contribution of SBS.POLKΔ to somatic mutations in cancer genomes.

The number of PCAWG whole genome mutation datasets analysed, and those showing more than 5% contribution of the polymerase kappa deficiency–specific SBS signature from Figure 1 (SBS.POLKΔ) when deconstructed to all COSMIC v3.1 SBS signatures plus SBS.POLKΔ. Samples are separated by tissue type.

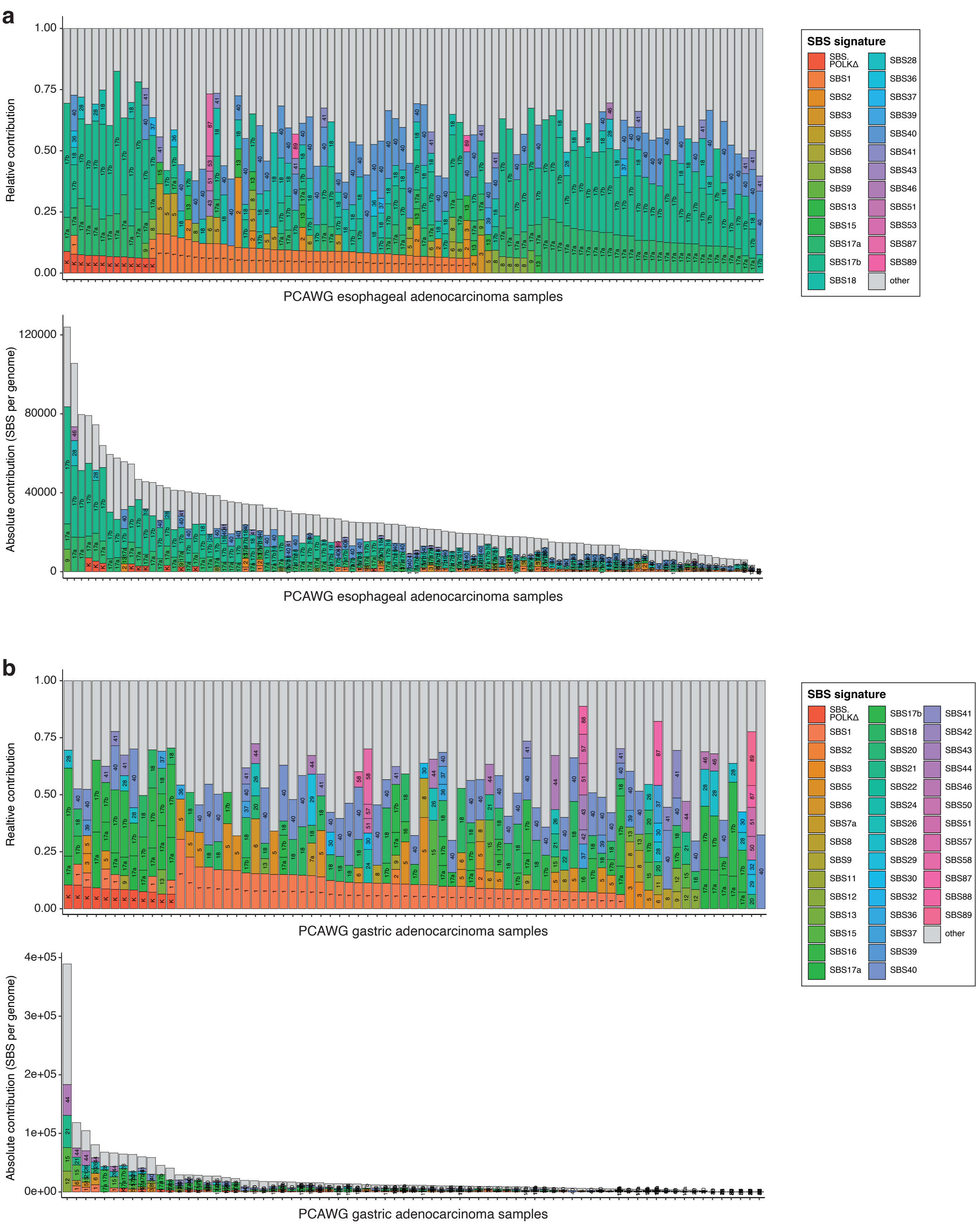

Supplementary Figure 6. The contribution of SBS.POLKΔ to somatic mutations in individual cancer genomes.

Deconstruction of PCAWG whole genome mutation datasets from esophageal (**a**) or gastric (**b**) cancers with COSMIC version 3.1 SBS signatures plus the polymerase kappa deficiency–specific SBS signature from Figure 1 (SBS.POLKΔ, labelled K). Signatures labelled 'other' contributed less than 6% each.

Supplementary Table 1: List of cell lines used in this study

Cell lines	Source
**DT40**	
WT	Buerstedde J.M., Reynaud C.A., Humphries E.H., Olson W., Ewert D.L., Weill J.C . (1990) Light chain gene conversion continues at high rate in an ALV-induced cell line. EMBO J 9, 921–927.
BRCA1-/-	Vandenberg C.J., Gergely F., Ong C.Y., Pace P., Mallery D.L., Hiom K., Patel K.J. (2003) BRCA1-independent ubiquitination of FANCD2. Mol Cell 12, 247–254.
BRCA2-/-	Qing Y., Yamazoe M., Hirota K., Dejsuphong D., Sakai W., Yamamoto K.N., Bishop D.K., Wu X.H., Takeda S. (2011) The epistatic relationship between BRCA2 and the other RAD51 mediators in homologous recombination. PloS Genet 7, e1002148.
REV1-/-	Simpson L., Sale J.E. (2003) Rev1 is essential for DNA damage tolerance and non-templated immunoglobulin gene mutation in a vertebrate cell line. EMBO J 7, 1654-1664.
POLH -/-	Kawamoto T., Araki K., Sonoda E., Yamashita Y.M., Harada K., Kikuchi K., Masutani C., Hanaoka F., Nozaki K., Hashimoto N., Takeda S. (2005) Dual Roles for DNA Polymerase η in Homologous DNA Recombination and Translesion DNA Synthesis. Molecular Cell 20, 793-799.
POLK -/-	Okada K., Sonoda E., Yamashita Y.M, Koyoshi S., Tateishi S., Yamaizumi M., Takata M., Ogawa O., Takeda S. (2002) Involvement of Vertebrate Polκ in Rad18-independent Postreplication Repair of UV Damage. J Biol Chem 277, 48690-48695.
53BP1-/-	Nakamura K., Sakai W., Kawamoto T., Bree R.T., Lowndes N.F., Takeda S., Taniguchi Y. (2006) Genetic dissection of vertebrate 53BP1: a major role in non-homologous end joining of DNA double strand breaks. DNA Repair 5, 741-749.
Ku70-/-	Takata M., Sasaki M.S., Sonoda E., Morrison C., Hashimoto M., Utsumi H., Yamaguchi-Iwai Y., Shinohara A., Takeda S. (1998) Homologous recombination and non-homologous end-joining pathways of DNA double-strand break repair have overlapping roles in the maintenance of chromosomal integrity in vertebrate cells. EMBO J 17, 5497-5508.
BRCA1-/- REV1-/-	this study
BRCA1-/- POLH -/-	this study
BRCA1-/- POLK -/-	this study
BRCA1-/- 53BP1-/-	this study
BRCA1-/- Ku70-/-	this study
**TK6**	
WT	Lorge, E., Moore, M.M., Clements, J., O'Donovan, M., Fellows, M.D., Honma, M., Kohara, A., Galloway, S., Armstrong, M.J., Thybaud, V. Gollapudi, B., Aardema, M.J., Tanir, J.Y. (2016) Standardized cell sources and recommendations for good cell culture practices in genotoxicity testing. Mutat Res 809, 1-15.
53BP1-/-	Sasanuma H., Tsuda M., Morimoto S., Saha L.K., Rahman M.M., Kiyooka Y., Fujiike H., Cherniack A.D., Itou J., Moreu E.C., Toi M., Nakada S., Tanaka H., Tsutsui K., Yamada S., Nussenzweig A., Takeda S. (2018) BRCA1 ensures genome integrity by eliminating estrogen-induced pathological topoisomerase II-DNA complexes. Proc Natl Acad Sci U S A. 115, E10642-E10651.

Supplementary Table 2: PCR primers used for the analysis of T antigen dependent in vitro replication

Replication efficiency	pUCQF0 forward	CTTCCTGTTTTTGCTCACCC
	pUCQF0 reverse	GTTCTTCGGGGCGAAAACT
	pUCQF3 forward	CTGAATGAAGCCATACCAAACG
	pUCQF3 reverse	TTTGCGCAACGTTGTTGCCATT
Outcome of lesion bypass, first round of PCR	forward	AGAACTCATATGGATCGAATTGTC
	reverse	GTTCTTCGGGGCGAAAAC
Outcome of lesion bypass, sequence-specific primer sets	sequence outcome	
		forward primers
	(TT)	CATATGGATCGAATTGTCCACCTCTT
	(GC)	TGGATCGAATTGTCCACCTCGCC
	(--)	TGGATCGAATTGTCCACCTCCCT
		forward primers
	(TT)	ATATGATCTGCATGGATCGATTT
	(GC)	ATGATCTGCATGGATCGAGCT
	(--)	TGATCTGCATGGATCGATAG

Supplementary Table 3: Suppliers and catalogue numbers of reagents

Reagent name	Supplier	Catalogue number
RPMI-1640 (Roswell Park Memorial Institute-1640 medium)	Life Technologies	21875091
FBS (Fetal Bovine Serum)	Life Technologies	10270106
Chicken serum	Sigma-Aldrich	C5405
2-mercaptoethanol	Sigma-Aldrich	444203
cisplatin	Sigma-Aldrich	C2538
MMS	Sigma-Aldrich	129925
Daunomycin	Sigma-Aldrich	D8809
Etoposide	Sigma-Aldrich	E1383
SN-38	Sigma-Aldrich	H0165
olaparib (AZD2281, KU0059436)	Selleckchem	S1060
Presto Blue Cell Viability Reagent	Thermo Fisher Scientific	A13261
E. coli BL21-CodonPlus (DE3)-RP *competent cell*	Agilent Technologies	230255
isopropyl-ß-D-1-thiogalactopyranoside (IPTG)	Sigma-Aldrich	I6758
Tris·HCl	VWR	103157P
NaCl	VWR	A2942
glycerol	Sigma-Aldrich	G7757
DTT	Sigma-Aldrich	10197777001
Triton X-100	Sigma-Aldrich	11332481001
Benzonase Nuclease	Millipore	71205
PreScission protease	Cytiva	27-0843-01
Resource Q chromatography column	Cytiva	17-1179-01
Superdex 200 Increase 10/300 GL	Cytiva	28990944
SV40 T antigen	EURx	E5800-02
HEPES	Sigma-Aldrich	H3375
$MgCl_2$	Sigma-Aldrich	208337
ATP	Thermo Fisher Scientific	PV3227
dNTP Set (100mM - dATP, dGTP, dTTP, dCTP)	Sigma-Aldrich	GE28-4065-51
Ribonucleoside triphosphate set (CTP, GTP, UTP)	Sigma-Aldrich	11277057001
Phosphocreatine disodium salt hydrate	Sigma-Aldrich	P7936
Creatine Phosphokinase from rabbit muscle	Sigma-Aldrich	C3755
SDS	Sigma-Aldrich	L4522
EDTA	Thermo Fisher Scientific	557628
Proteinase K	Sigma-Aldrich	P2308
Ammonium acetate	Sigma-Aldrich	1542
isopropanol	VWR	20842.312
ethanol	VWR	20821.321
DpnI	New England Biolabs	R0176L
Xceed qPCR SG 2x Mix Lo-ROX	Institute of Applied Biotechnologies	LPCR10501XL
anti-53BP1	Santa Cruz Biotechnology	sc-517281
anti-α-tubulin	Sigma-Aldrich	T6199
anti-chicken IgM-FITC	Bethyl Laboratories	A30-102F
