# Supplementary material for: BRCA1 deficiency specific base substitution mutagenesis is dependent on translesion synthesis and regulated by 53BP1
Source: Nat Commun. 2022 Jan 11;13:226. doi: 10.1038/s41467-021-27872-7 (PMC8752635; doi:10.1038/s41467-021-27872-7)
Supplement: Supplementary file 3 — Description of additional Supplementary File [file 41467_2021_27872_MOESM3_ESM.pdf]

Descriptions of additional supplementary data files

Supplementary Data 1: Sequencing information and statistics for all whole genome sequenced samples presented in this study.

Supplementary Data 2: Catalog of all detected and post-filtered single base substitution (SBS) mutations.

Supplementary Data 3: Catalog of all detected and post-filtered insertion and deletion mutations.

Supplementary Data 4: Summary and classification of all genetic alterations found in the whole genome sequenced cell line samples.

Supplementary Data 5: Nucleotide triplet contributions of de novo SBS signatures identified in this study.

Supplementary Data 6: Comparison of de novo SBS signatures to COSMIC v3.2 SBS signatures. Cosine similarity values are shown.
