# Supplementary material for: BRCA1 deficiency specific base substitution mutagenesis is dependent on translesion synthesis and regulated by 53BP1
Source: Nat Commun. 2022 Jan 11;13:226. doi: 10.1038/s41467-021-27872-7 (PMC8752635; doi:10.1038/s41467-021-27872-7)
Supplement: Supplementary file 10 — Reporting Summary [file 41467_2021_27872_MOESM10_ESM.pdf]

nature portfolio

Corresponding author(s): David Szuts

Last updated by author(s): Sep 19, 2021

Reporting Summary

Nature Portfolio wishes to improve the reproducibility of the work that we publish. This form provides structure for consistency and transparency in reporting. For further information on Nature Portfolio policies, see our Editorial Policies and the Editorial Policy Checklist.

Statistics

For all statistical analyses, confirm that the following items are present in the figure legend, table legend, main text, or Methods section.

n/a	Confirmed	
☐	☒	The exact sample size (*n*) for each experimental group/condition, given as a discrete number and unit of measurement
☐	☒	A statement on whether measurements were taken from distinct samples or whether the same sample was measured repeatedly
☐	☒	The statistical test(s) used AND whether they are one- or two-sided<br>*Only common tests should be described solely by name; describe more complex techniques in the Methods section.*
☒	☐	A description of all covariates tested
☒	☐	A description of any assumptions or corrections, such as tests of normality and adjustment for multiple comparisons
☐	☒	A full description of the statistical parameters including central tendency (e.g. means) or other basic estimates (e.g. regression coefficient) AND variation (e.g. standard deviation) or associated estimates of uncertainty (e.g. confidence intervals)
☐	☒	For null hypothesis testing, the test statistic (e.g. *F*, *t*, *r*) with confidence intervals, effect sizes, degrees of freedom and *P* value noted<br>*Give P values as exact values whenever suitable.*
☒	☐	For Bayesian analysis, information on the choice of priors and Markov chain Monte Carlo settings
☒	☐	For hierarchical and complex designs, identification of the appropriate level for tests and full reporting of outcomes
☒	☐	Estimates of effect sizes (e.g. Cohen's *d*, Pearson's *r*), indicating how they were calculated

Our web collection on statistics for biologists contains articles on many of the points above.

Software and code

Policy information about availability of computer code

Data collection	Bio-Rad CFX Manager 3.0 was used for the collection of quantitative PCR data, and Bio-Rad Image Lab 4.1 for the collection of Western blot images.
Data analysis	The following software was used for data analysis:<br>FastQC v0.11.8<br>bwa 0.7.17-r1194-dirty<br>samblaster 0.1.24<br>samtools 1.8-35-g22caad0<br>GATK v3.8-0-ge9d806836<br>R 3.8.1 with the following packages:<br>tidyverse 1.3.1<br>superheat 0.1.0<br>Rsamtools 2.8.0<br>BSgenome.Ggallus.UCSC.galGal4 1.4.0<br>GenomicRanges 1.44.0<br>MutationalPatterns 3.0.1<br>deconstructSigs 1.8.0<br><br>In addition, custom scripts used for the analysis of immunoglobulin mutation data are deposited at https://github.com/szutsgroup/BRCA1_TLS_mutagenesis

For manuscripts utilizing custom algorithms or software that are central to the research but not yet described in published literature, software must be made available to editors and reviewers. We strongly encourage code deposition in a community repository (e.g. GitHub). See the Nature Portfolio guidelines for submitting code & software for further information.

Data

Policy information about availability of data

All manuscripts must include a data availability statement. This statement should provide the following information, where applicable:

- Accession codes, unique identifiers, or web links for publicly available datasets
- A description of any restrictions on data availability
- For clinical datasets or third party data, please ensure that the statement adheres to our policy

Source data are provided with this paper. Mutation lists and further source data for mutational analyses are provided as Supplementary Data. Raw whole genome sequence data generated in this study is available from the European Nucleotide Archive under study accession number PRJEB44196 [https://www.ebi.ac.uk/ena/browser/view/PRJEB44196].

Field-specific reporting

Please select the one below that is the best fit for your research. If you are not sure, read the appropriate sections before making your selection.

☒ Life sciences ☐ Behavioural & social sciences ☐ Ecological, evolutionary & environmental sciences

For a reference copy of the document with all sections, see nature.com/documents/nr-reporting-summary-flat.pdf

Life sciences study design

All studies must disclose on these points even when the disclosure is negative.

Sample size	Sample sizes were almost uniformly n=3, i.e. three independent clones were subjected to whole genome sequencing. This sample size was chosen due to cost considerations. n=3 was sufficient to show significant differences upon diverse biological conditions in mutation numbers in our previous, relevant publications (upon HR deficiency: Zamborszky et al., 2017, upon cisplatin treatment: Szikriszt et al., 2016; these publications are cited in the manuscript) therefore it was appropriate to use the same sample sizes for the presented related experiments. For experiments presented in figures 4, 5 and 6 the sample number was also n=3. The standard deviations and the expected effects were not known in advance, therefore this sample number was chosen as a uniform standard in advance and only those differences were reported as significant which had $p<0.05$ with two-sided unpaired t-tests.
Data exclusions	No data were excluded, all sequenced genomes were used for analyses.
Replication	The experimental findings were reproducible. To confirm the reproducibility of the long term mutagenesis experiments, we sequenced 2 batches of mock-treated BRCA1 mutant cell clones several years apart, and used all six samples for the analyses. Whole genome sequencing based experiments with all other genotypes were performed on a single occasion with three parallel biological replicates (separate cell clones were sequenced). Immunoglobulin hypermutation assays in figure 4 were all performed in parallel, with three biological replicates. Experiments in figures 5 and 6 were performed independently on n=3 occasions.
Randomization	Our study did not involve allocation of samples to different experimental groups. Randomisation is therefore not relevant. Experimental groups were mostly based on cell line identities, which were known. When treated and not treated populations or cell clones were compared, these were based on the bulk separation of an original starting population, not on the allocation of individual samples to experimental groups.
Blinding	All bioinformatics analyses (mutation detection) are exactly reproducible, therefore blinding was not necessary. Nevertheless, the software methods do not require an input about the identity of the samples (e.g. which sequenced genomes have the same genotype), therefore in essence the analyses were blinded. Cytotoxicity measurements were performed by a pipetting robot and automated plate reader, therefore again there was no subjective involvement of the investigator during data collection. Cytotoxicity measurements did not involve data analysis beyond the presentation of raw and normalised data points, so blinding was not applicable. The analysis of the outcome of in vitro replication experiments (figure 6) by quantitative PCR was performed in a blinded manner by a different investigator from the one who performed the replication reaction.

Reporting for specific materials, systems and methods

We require information from authors about some types of materials, experimental systems and methods used in many studies. Here, indicate whether each material, system or method listed is relevant to your study. If you are not sure if a list item applies to your research, read the appropriate section before selecting a response.

Materials & experimental systems

n/a	Involved in the study
☐	☒ Antibodies
☐	☒ Eukaryotic cell lines
☒	☐ Palaeontology and archaeology
☒	☐ Animals and other organisms
☒	☐ Human research participants
☒	☐ Clinical data
☒	☐ Dual use research of concern

Methods

n/a	Involved in the study
☒	☐ ChIP-seq
☒	☐ Flow cytometry
☒	☐ MRI-based neuroimaging

Antibodies

Antibodies used

Anti-chicken IgM-FITC conjugate (1:100, Bethyl Laboratories, A30-102F), anti-53BP1 (1:500, sc-517281, clone 6B3E10, Santa Cruz Biotechnology), anti-α-tubulin (1:2000, T6199, Sigma-Aldrich)

Validation

The anti-53BP1 antibody recognised a strong band in human cell extracts above 200 kDa as described by the manufacturer [https://www.scbt.com/p/53bp1-antibody-6b3e10] - and also a weaker band lowed down. Both bands disappeared entirely in extracts made from isogenic 53BP1-/- deficient human TK6 cells, which validates them as specific. The anti-alpha-tubulin antibody recognised a single strong approx. 50kDa band in human cell extracts, exactly as shown for various human cell lines on the manufacturer's web site [https://www.sigmaaldrich.com/HU/en/product/sigma/t6199].

Eukaryotic cell lines

Policy information about cell lines

Cell line source(s)

DT40 cell lines:
WT Buerstedde J.M., Reynaud C.A., Humphries E.H., Olson W., Ewert D.L., Weill J.C . (1990) Light chain gene conversion continues at high rate in an ALV-induced cell line. EMBO J 9, 921–927.
BRCA1-/- Vandenberg C.J., Gergely F., Ong C.Y., Pace P., Mallery D.L., Hiom K., Patel K.J. (2003) BRCA1-independent ubiquitination of FANCD2. Mol Cell 12, 247–254.
BRCA2-/- Qing Y., Yamazoe M., Hirota K., Dejsuphong D., Sakai W., Yamamoto K.N., Bishop D.K., Wu X.H., Takeda S. (2011) The epistatic relationship between BRCA2 and the other RAD51 mediators in homologous recombination. PloS Genet 7, e1002148.
REV1-/- Simpson L., Sale J.E. (2003) Rev1 is essential for DNA damage tolerance and non-templated immunoglobulin gene mutation in a vertebrate cell line. EMBO J 7, 1654-1664.
POLH -/- Kawamoto T., Araki K., Sonoda E., Yamashita Y.M., Harada K., Kikuchi K., Masutani C., Hanaoka F., Nozaki K., Hashimoto N., Takeda S. (2005) Dual Roles for DNA Polymerase η in Homologous DNA Recombination and Translesion DNA Synthesis. Molecular Cell 20, 793-799.
POLK -/- Okada K., Sonoda E., Yamashita Y.M, Koyoshi S., Tateishi S., Yamaizumi M., Takata M., Ogawa O., Takeda S. (2002) Involvement of Vertebrate Polκ in Rad18-independent Postreplication Repair of UV Damage. J Biol Chem 277, 48690-48695.
53BP1-/- Nakamura K., Sakai W., Kawamoto T., Bree R.T., Lowndes N.F., Takeda S., Taniguchi Y. (2006) Genetic dissection of vertebrate 53BP1: a major role in non-homologous end joining of DNA double strand breaks. DNA Repair 5, 741-749.
Ku70-/- Takata M., Sasaki M.S., Sonoda E., Morrison C., Hashimoto M., Utsumi H., Yamaguchi-Iwai Y., Shinohara A., Takeda S. (1998) Homologous recombination and non-homologous end-joining pathways of DNA double-strand break repair have overlapping roles in the maintenance of chromosomal integrity in vertebrate cells. EMBO J 17, 5497-5508.
BRCA1-/- REV1-/- this study
BRCA1-/- POLH -/- this study
BRCA1-/- POLK -/- this study
BRCA1-/- 53BP1-/- this study
BRCA1-/- Ku70-/- this study

TK6 cell lines:
WT Lorge, E., Moore, M.M., Clements, J., O'Donovan, M., Fellows, M.D., Honma, M., Kohara, A., Galloway, S., Armstrong, M.J., Thybaud, V. Gollapudi, B., Aardema, M.J., Tanir, J.Y. (2016) Standardized cell sources and recommendations for good cell culture practices in genotoxicity testing. Mutat Res 809, 1-15.
53BP1-/- Sasanuma H., Tsuda M., Morimoto S., Saha L.K., Rahman M.M., Kiyooka Y., Fujiike H., Cherniack A.D., Itou J., Moreu E.C., Toi M., Nakada S., Tanaka H., Tsutsui K., Yamada S., Nussenzweig A., Takeda S. (2018) BRCA1 ensures genome integrity by eliminating estrogen-induced pathological topoisomerase II-DNA complexes. Proc Natl Acad Sci U S A. 115, E10642-E10651.

Authentication

All cell lines were authenticated using the whole genome sequencing data.

Mycoplasma contamination

The cell lines were tested and found negative for mycoplasma contamination.

Commonly misidentified lines
(See ICLAC register)

No such lines were used.
